# SRflow: Deep learning based super-resolution of 4D-flow MRI data

**DOI:** 10.3389/frai.2022.928181

**Published:** 2022-08-12

**Authors:** Suprosanna Shit, Judith Zimmermann, Ivan Ezhov, Johannes C. Paetzold, Augusto F. Sanches, Carolin Pirkl, Bjoern H. Menze

**Affiliations:** ^1^Department of Informatics, Technical University of Munich, Munich, Germany; ^2^Department of Quantitative Biomedicine, University of Zurich, Zurich, Switzerland; ^3^Institute of Neuroradiology, University Hospital LMU Munich, Munich, Germany

**Keywords:** 4D-flow MRI, residual learning, flow super-resolution, cerebrovascular flow, flow quantification

## Abstract

Exploiting 4D-flow magnetic resonance imaging (MRI) data to quantify hemodynamics requires an adequate spatio-temporal vector field resolution at a low noise level. To address this challenge, we provide a learned solution to super-resolve *in vivo* 4D-flow MRI data at a post-processing level. We propose a deep convolutional neural network (CNN) that learns the inter-scale relationship of the velocity vector map and leverages an efficient residual learning scheme to make it computationally feasible. A novel, direction-sensitive, and robust loss function is crucial to learning vector-field data. We present a detailed comparative study between the proposed super-resolution and the conventional cubic B-spline based vector-field super-resolution. Our method improves the peak-velocity to noise ratio of the flow field by 10 and 30% for *in vivo* cardiovascular and cerebrovascular data, respectively, for 4 × super-resolution over the state-of-the-art cubic B-spline. Significantly, our method offers 10x faster inference over the cubic B-spline. The proposed approach for super-resolution of 4D-flow data would potentially improve the subsequent calculation of hemodynamic quantities.

## 1. Introduction

Assessing quantitative hemodynamic metrics is crucial in diagnosing and managing flow-mediated vascular pathologies. For example, monitoring wall shear stress along the aortic vessel wall supports diagnostic assessment in patients with bicuspid aortic valves (Guzzardi et al., 2015; Garcia et al., 2019); alteration in pressure distribution (Leidenberger et al., 2020) is observed in Marfan disease. Similarly, local characterization of the vortex core pattern can assist in rapture risk estimation of the vascular aneurysm (Futami et al., 2019). 4D-flow magnetic resonance imaging (4D-flow MRI) (Markl et al., 2012) provides spatiotemporally resolved velocity vector maps of coherent blood flow through vascular structures. In applications mentioned above, 4D-flow MRI serves as a basis for quantifying flow parameters and patterns non-invasively.

Accurate computation of image-based quantitative hemodynamic metrics from 4D-flow MRI is limited by the trade-offs between spatiotemporal resolution, signal-to-noise ratio, and the clinically acceptable *in vivo* acquisition duration. In particular, low spatial resolution at near-wall points hamper the numerical estimation of spatial derivatives of the three-dimensional vector field (Petersson et al., 2012). Furthermore, sometimes image registration is required to standardize the image space for comparison purposes (Cibis et al., 2017), which also involves isotropic resampling or super-resolution of the 4D-flow data. Moreover, clinically important qualitative visualization, such as streamlines (Cebral et al., 2011) and vortex core line (Byrne et al., 2014) delineation, relies on improved spatial resolution. Therefore, having access to high-resolution 4D-flow MRI image data is critical to infer hemodynamic metrics reliably and using computational routines for improving the image data after acquisition has become an indispensable step in processing these data. This brings to the generations of efficient acquisition algorithms and hardware acceleration, such as parallel imaging (Stankovic et al., 2014), non-Cartesian trajectories (Markl et al., 2012), k-t SENSE (Tsao et al., 2003), k-t GRAPPA (Breuer et al., 2005), which enabled 4D-flow MRI. In addition, several algorithms have emerged over time, building on this accelerated acquisition to enhance the measured 4D-flow MRI. In this direction, there are two parallel streams of research in the context of MRI super-resolution: 1) Compressed sensing: MRI super-resolution by improving high-frequency components from the k-space (Santelli et al., 2016; Ma et al., 2019) and 2) Single-volume MRI super-resolution: acquiring conventional MRI at low spatiotemporal resolution and retrospectively super-resolve data at the image level (Ferdian et al., 2020) as post-processing.

Previous works based on MRI image quality enhancement from k-space were focused toward velocity-field denoising (Ong et al., 2015), divergence reduction (Mura et al., 2016), intravoxel dephasing (Rutkowski et al., 2021), and streamline denoising (Callaghan and Grieve, 2017). In contrast, super-resolution as a post-processing step in the image space is more versatile and applicable to any collection of images acquired by differing sequences or MRI scanners, agnostic to the specifics of the k-space sampling. Once we have the reconstructed MRI images in the form of DICOM or NIFTI, super-resolution in image space is an efficient and hustle-free plug-and-play feature. As such, it is not a competing but a complementary and independent field of work that is of particular relevance when dealing with large and inhomogeneous multi-centric data sets or when access to original k-space recordings is not available. In spite of an abundance of conceptually related machine learning-based techniques for video, super-resolution (Chu et al., 2018) and optical-flow estimation (Liu et al., 2019), (that has not been used for 4D-flow MRI super-resolution, though) the data-driven reconstruction in image space remains under-explored in 4D-flow MRI, which is a commonly used method either rely on 4D cubic spline (Stalder et al., 2008; Dyverfeldt et al., 2014) or sinc (Bernstein et al., 2001) interpolation. In this work, we will focus on adapting deep learning-based super-resolution to the specific requirements of 4D-flow interpolation, leveraging prior knowledge of flow fields from prior observations. At the same time, we identify that the loss function is crucial for this translation and offers a novel loss well-adapted to velocity fields.

### 1.1. Prior work on super-resolution in image space

Super-resolution is a well-studied topic in computer vision, where high-resolution images are reconstructed from low-resolution images. Recently, deep neural networks based on super-resolution (Bhowmik et al., 2018) have become popular due to their high accuracy and fast processing time. Dong et al. (2016) first introduced a fully convolutional network for super-resolution. Most of the subsequent super-resolution approaches rely on residual learning, where we predict the fine detail using a convolutional network and add them with coarse upsampled images (i.e., cubic spline). Two distinct approaches in residual learning for super-resolution evolved in recent times: a) upsample in the beginning and then extract fine details from it using residual learning (Kim et al., 2016) and b) extract powerful image features from the low-resolution image and add them with the upsampled image at the end (Lim et al., 2017). The former enjoys extra performance improvement, while the latter is more efficient regarding the computational budget. Recently, channel widening before activation in the residual branch has been proposed (Yu et al., 2018), which not only helps shallow features to propagate easily into deeper layers but also reduces the network complexity. Zhang et al. (2018) has proposed a residual in residual architecture for a very deep network with a channel attention layer, which exploits non-linear interaction between global channel statistics to scale individual features.

### 1.2. Prior work on MRI super-resolution and challenges

Volumetric MRI super-resolution (Pham et al., 2017; Lyu et al., 2020) in image space is analogous to the 2D counterpart. However, the challenge lies in designing memory- and computation-efficient methods suitable for 3D volume that can be trained on a limited amount of training data. This problem is exaggerated for 3D vector-valued data, requiring new approaches to learn the inter-scale transformation effectively. Previously, in an attempt to mitigate the 3D computational complexity, a variation of residual learning called densely connected convolutional network has been adopted in MRI super-resolution by Chen et al. (2018). Often super-resolution is interpreted as a texture synthesis problem using adversarial learning (Sánchez and Vilaplana, 2018). Although adversarial learning produces perceptually high-quality images (Xie et al., 2018), it fails to achieve superior reconstruction metrics compared to non-adversarial learning, which is of main interest in the case of 4D-flow MRI to accurately compute the velocities and their spatial derivatives. Hence, we are not considering adversarial learning. Previously, data-driven super-resolution approaches explored other MRI modalities, such as super-resolution of temporal perfusion MRI (Meurée et al., 2019) and vector field super-resolution problem of diffusion MRI (Tanno et al., 2017; Albay et al., 2018). Note that these methods either rely on 2D slice-wise super-resolution or individual channel-wise super-resolution in 3D. Ferdian et al. (2020) proposed a residual network to super resolve 4D Flow MRI. Although they also used computational fluid dynamic (CFD) to mitigate the shortcomings of noisy *in vivo* data, their method relies on the magnitude image alongside the velocity image. For CFD, it is hard to simulate a magnitude image due to the unknown relationship between the simulated velocity field and the magnitude image intensities. Fathi et al. (2020) proposed physics-informed deep learning to super resolve patient-specific flow. However, they need to retrain their model for each new patient since the physics-informed model does not generalize over the computation domain, i.e., the vessel geometry in this case.

### 1.3. Related work on 4D-flow MRI and CFD

Several classical approaches have been applied to improve the spatial resolution of 4D-fLow MRI, such as ridge-regression (Bakhshinejad et al., 2017) and Lasso regression (Fathi et al., 2018). Recently, Rutkowski et al. (2021) proposed a machine learning-based solution to merge the CFD and MRI data. Flow data assimilation is another active research field, where the reduced-order Kalman filter (Habibi et al., 2021) and local ensemble Kalman filter (Gaidzik et al., 2019, 2021) have been used. Incorporating CFD simulation using interior-point optimization framework (Töger et al., 2020) and lattice Boltzmann-based topology optimization (Klemens et al., 2020). While these works attempted to improve 4D-flow MRI by merging CFD data, it requires expensive CFD simulation for each new acquisition. Hence, we look for an alternative road where we can learn a model using both CFD and *in vivo* 4D-flow MRI data, and the learned solution can be used out of the box for any newly acquired data without any further CFD simulation.

Along this line, we aim to find an elegant solution to learn a scalable non-linear mapping from coarse- to fine-scale spatial velocity field. Further, three channels of 4D-flow MRI together represent the flow direction in 3D and should be treated as a joint interpolation problem compared to earlier approaches on scalar volumes (Chen et al., 2018; Sánchez and Vilaplana, 2018). Moreover, the commonly used ℓ_2_ loss is sub-optimal for 4D-flow MRI because of the non-Gaussian (Gudbjartsson and Patz, 1995) noise distribution and does not prioritize the direction of point-wise velocity fields. Previously, direction-sensitive loss functions, such as cosine similarity, have been explored in text processing (Li and Han, 2013) and face recognition (Nguyen and Bai, 2010). Since in 4D-flow MRI, the flow direction consistency is important for all subsequent applications, we identify it as a crucial aspect and propose including it in a novel *mutually-projected* ℓ_1_ loss function.

### 1.4. Our contribution

In summary, our contributions are as follows:

- We propose a novel and memory-efficient end-to-end convolutional neural network architecture, which learns the non-linear relationship between fine- and coarse-scale velocity fields and achieves super-resolution of the velocity field. Moreover, it applies to 4D-flow data irrespective of the scanner-specific constraints and access to the k-space information.
- We introduce a novel, robust, and direction-dependent cost function referred to as *mutually projected* ℓ_1_. We investigate its effect on the proposed network compared to the standard ℓ_1_ loss function.
- We further validate our method on *in vivo* 4D flow MRI datasets of two anatomical regions, namely: a) an internal carotid artery (ICA) brain aneurysm (Cerebrovascular data) and b) whole heart and great vessel (Cardiovascular data) that were acquired with different MRI scanners and at different imaging centers. This assesses the generalizability of the proposed method.

## 2. Materials and methods

In this section, we describe in detail the proposed learning-based method (Section 2.1). Subsequently, we describe the proposed robust loss function (Section 2.2) along with its implementation details (Section 2.3).

### 2.1. Network architecture

4D-flow MRI provides time-resolved 3D blood flow velocity maps over a single cardiac cycle. Our work focuses on super-resolving along the spatial dimensions and treats each temporal image frame as an independent sample. Let us denote the low resolution velocity field and high resolution velocity field as **u** and **U** respectively, where $\text{u}\in R^{H\times W\times D\times 3}$, $\text{U}\in R^{sH\times sW\times sD\times 3}$, *H, W, D* are the spatial dimensions, and *s* is the factor of upscaling (*s* = 2, 3or4). We are interested in learning a supervised data-driven mapping function from **u**→**U** from the input-output pairs ${\left\{{\text{u}}_{i},{\text{U}}_{i}\right\}}_{i=1}^{n}$. Since three channels denote three velocity components are highly correlated, we opted for a three-channel volumetric super-resolution instead of individually super-resolving each velocity component. To reduce the computational overhead, we naturally opt to extract a rich feature from the low-resolution vector field using residual learning and add the predicted fine details with upsampled vector fields to reconstruct high-resolution velocity fields.

#### 2.1.1. Building blocks

**Figure 1:**
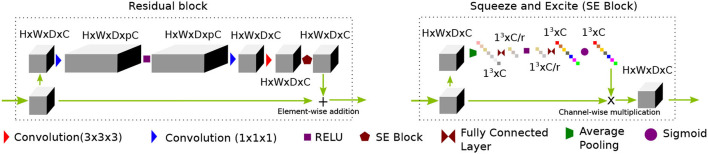
A proposed residual block consists of a sequence of channel widening, convolution followed by activation, channel squeeze and excitation (SE) block. While the channel widening by a factor of *p* before activation helps to reduce network parameters, the SE block promotes diverse feature distribution by compressing interim features by a factor of *r*.

**Figure 2:**
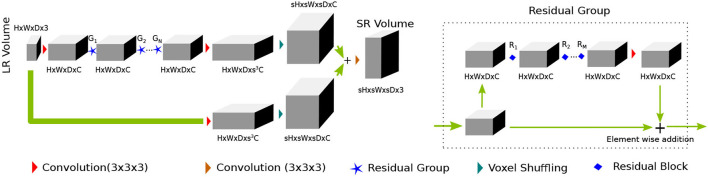
PA proposed SRflow has a series of residual groups made of residual blocks, as shown in Figure 1. The residual grouping strategy maximally leverages residual learning by exploiting hierarchical skip connections.

In recent times (Yu et al., 2018), wide-activation before the convolution operation in the residual branch proved to be an efficient strategy to reduce model complexity without sacrificing super-resolution performance. We first extend the idea of having widely activated residual blocks in 3D. Henceforth, we will call this network WDSR-3D and use it as a baseline method for evaluation purposes. We keep the weight normalization as was proposed in Yu et al. (2018). We incorporate a sequence of residual groups, which effectively accelerates the learning of deep networks (Zhang et al., 2018). Since channel expansion with a 1 × 1 × 1 convolution kernel is applied, new channels are different linear combinations of the previous layer channel without any spatial feature propagating in the channel dimension. Thus, information in the new channels carries redundant information. We argue that although this redundancy gives multiple paths for the gradient to propagate easily throughout the residual blocks, they often share similar information. The recently introduced squeeze and excitation (SE) (Hu et al., 2018) block helps to inject useful cross-channel diversity in the residual features. Hence, we leverage the feature diversity of an SE block to re-calibrate the channel features. This is depicted in Figure 1. We will refer to this modified architecture as SRflow in the subsequent discussion, which is depicted in Figure 2.

#### 2.1.2. SRflow

The first convolution layer transforms the input to *C* channel feature maps. After first convolution, it goes through the *N* number of residual groups **G**_**1**_, **G**_**2**_, ⋯ , **G**_**N**_. Each of the residual group consists of *M* number of **R**_**1**_, **R**_**2**_, ⋯ , **R**_**M**_ residual blocks. The deep features and the input goes through their respective feature refining convolution layer, which transforms channels *C*→*s*^3^*C*. We use voxel shuffling layer [3D pixel shuffling (Shi et al., 2016) layer] to rearrange features from channel dimension to increase spatial dimension *sH* × *sW* × *sD* × *C*. The final convolution layer merges fine details with the coarse up-scaled branch and reconstruct super-resolved volume of size *sH* × *sW* × *sD* × 3.

### 2.2. Robust loss function

4D-flow MRI data acquired in clinical settings are sparse in both space and time, and they can be easily corrupted by noise. Since noise in 4D-flow MRI is not Gaussian (Gudbjartsson and Patz, 1995), it is sub-optimal to use an ℓ_2_ norm on the error in our task. Thus, a robust cost function is needed for obtaining accurate estimates of the super-resolved flow. Let us denote the reference velocity as ${\left\{{\text{u}}_{i}\right\}}_{i=1}^{n}|{\text{u}}_{i}\in R^{3}$ and the estimated velocity as ${\left\{{\text{v}}_{i}\right\}}_{i=1}^{n}|{\text{v}}_{i}\in R^{3}$. The most commonly used robust loss function is ℓ_1_ loss. For *n* number of samples, it is defined as


(1) $$\begin{array}{r} J_{\ell_{1}}=\frac{1}{n}\overset{n}{\underset{i=1}{\sum }}||{\text{v}}_{i}-{\text{u}}_{i}|{|}_{1} \end{array}$$

#### 2.2.1. Mutually projected ℓ_1_ loss

**Figure 3:**
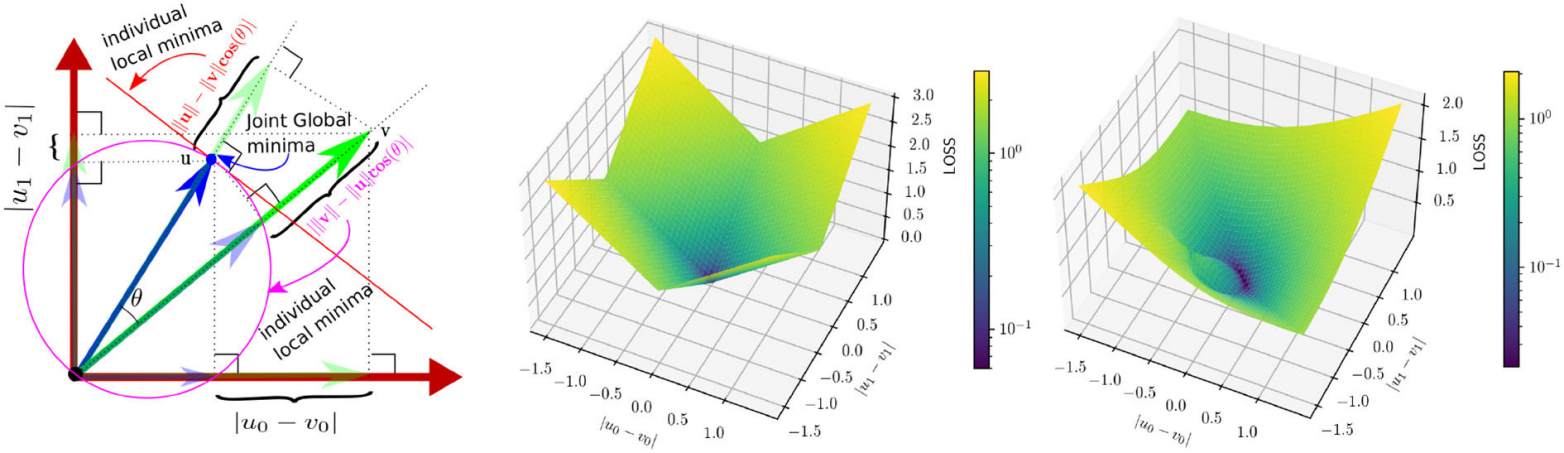
Left: A 2D delineation of *mutually projected* ℓ_1_ (mp-ℓ_1_) error. The reference and the predicted vectors are **u** = [*u*_0_, *u*_1_] and **v** = [*v*_0_, *v*_1_], respectively. Individual local minimas of $J_{1}\left(\text{v};\text{u}\right)$ and $J_{2}\left(\text{v};\text{u}\right)$ w.r.t **v** are shown in red line and purple circle, respectively. However the common minima of the two losses are unique and are as same as the reference. Middle: A description of the ℓ_1_ error surface at **u** = [0.5, 0.5]. Right: A description of the *mp*−ℓ_1_ error surface for the same **u** and α = β = 0.5, which has a direction sensitivity based on the value of **u**.

ℓ_1_ loss penalizes the estimation error equally, irrespective of the reference vector direction. Neighboring voxels tend to have a different correlation in magnitude and direction based on local blood vessel geometry and the global flow direction. Because of this, we argue that a magnitude/direction disentanglement in the loss would benefit the network to arrive at a better trade-off between the accuracy of magnitude and direction estimation under noisy circumstances. Also, errors in magnitude and direction are very different in the value range across the spatial location, making it difficult to find optimal weight in the case of a weighted loss function. To overcome this, we propose to incorporate a directional sensitivity for the reference velocity in the loss function. Specifically, we introduce *mutually projected* ℓ_1_ (mp-ℓ_1_) error (c.f. Figure 3). The projected ℓ_1_ error of **v** on **u** is given by


(2) $$\begin{array}{r} J_{1}\left(\text{v};\text{u}\right)=|||\text{u}||-||\text{v}||\cos\left(\theta \right)| \end{array}$$

Where θ is the angle between **u** and **v**. The local minima of $J_{1}\left(\text{v};\text{u}\right)$ is the orthogonal subspace of **u**. Similarly, the projected ℓ_1_ error of **u** on **v** is given by


(3) $$\begin{array}{r} J_{2}\left(\text{v};\text{u}\right)=|||\text{v}||-||\text{u}||\cos\left(\theta \right)| \end{array}$$

The local minima of $J_{2}\left(\text{v};\text{u}\right)$ is the sphere centered at **u**/2 with radius ||**u**||/2 excluding the origin **0**. We take a convex linear combination of $J_{1}$ and $J_{2}$ to construct the $J_{\text{mp-}\ell_{1}}$ loss


(4) $$\begin{array}{r} J_{\text{mp-}\ell_{1}}=\frac{1}{n}\overset{n}{\underset{i=1}{\sum }}\left(\alpha J_{1}\left({\text{v}}_{i};{\text{u}}_{i}\right)+\beta J_{2}\left({\text{v}}_{i};{\text{u}}_{i}\right)\right) \end{array}$$

where [α+β = 1:0 < α, β <1]

Note that both $J_{1}$ and $J_{2}$ independently have two different solution spaces (cf Figure 3-left); however, $J_{\text{mp-}\ell_{1}}$ has a unique local minima, which is also the global minima achieved under the condition of **v** = **u**. Figure 3 explains mp-ℓ_1_ in 2D scenario. The same interpretation holds in a higher dimension with a hyper-sphere and a hyper-plane instead of a circle and a line. Unlike ℓ_1_ loss, mp-ℓ_1_ has directional sensitivity depending on the value of **u**, which helps the pointwise error to adapt locally near the minima.

#### 2.2.2. Combined loss

In our experiment, we find that the combination of ℓ_1_ and mp-ℓ_1_ losses helps to achieve the best performance in terms of training loss and validation PVNR. The complete loss function is as follows


(5) $$\begin{array}{r} J_{opt}=\lambda_{\ell_{1}}J_{\ell_{1}}+\lambda_{\text{mp-}\ell_{1}}J_{\text{mp-}\ell_{1}} \end{array}$$

where λ_ℓ_1__ and λ_mp-_ℓ__1__ are two weight parameters.

### 2.3. Implementation details

We implement our model in PyTorch. In the network architecture, we use *N* = 4and*M* = 2. We expand the features by *p* = 32 times for the wide activation, and for the SE block, we use *r* = 8. For loss function, we select α = β = 0.5 and λ_ℓ_1__ = λ_mp-_ℓ__1__ = 1. We select the learning rate at 10^−3^ and use the ADAM optimizer for all of our experiments. We train each model for 200 epochs with a learning rate decay of 0.9 after every 10 epochs with a batch size of 4 in a Quadro P6000 GPU. The best model is chosen based on the validation peak velocity-to-noise ratio (PVNR).

### 2.4. Datasets

**Figure 4:**
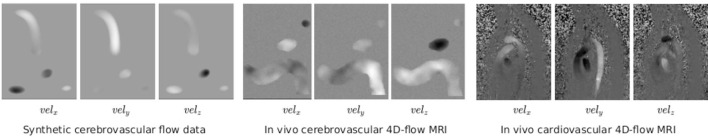
Typical examples of three data sets used in our experiments.

To study how well our model generalizes in practice, we chose two different vascular regions, which are also of numerous clinical relevance (Amili et al., 2018; Garcia et al., 2019). Importantly, these two datasets are obtained from two different scanners. The dataset consists of three different sets of flow data; a) **Synthetic Cerebrovascular Data:** CFD simulated flow data of cerebral aneurysm, b) ***In vivo* Cerebrovascular Data:** *in vivo* 4D-flow MRI of ICA aneurysm, and c) ***In vivo* Cardiovascular Data:** *in vivo* 4D-flow MRI of the whole heart and great vessels. Exemplary samples from the datasets have been shown in Figure 4.

#### 2.4.1. Synthetic cerebrovascular data

We obtain patient-specific cerebrovascular aneurysms (N = 6) in ICA geometries from 3D rotational angiograms. We segment the blood vessel geometries from the computed tomography using the MITK v2018.4. We generate the triangulated mesh using ICEM CFD v.19 (ANSYS Inc). We model the blood flow as an unsteady Newtonian flow and solve the Navier-Stokes equations using the finite volume-based OpenFOAM-v3.0. We impose the inlet patient-specific flow boundary conditions extracted from 2D phase-contrast MRI and a zero pressure condition at the outlet. All the vascular walls are assumed rigid. We use a second-order upwind scheme for the convective terms and a semi-implicit method for pressure-linked equations. We employ an algebraic multi-grid-based solver for high-precision simulation. We simulate the blood flow with the following parameters: viscosity 0.0032 Pa·s; density 1,050 kg/m^3^ (Brindise et al., 2019).

#### 2.4.2. *In vivo* 4D-flow MRI data

A total number of 24 *in vivo* 4D-flow MRI data sets are included: Cardiovascular data of healthy subjects covering the whole heart (*N* = 10) or thoracic aorta only (*N* = 11); and cerebrovascular data of patients with ICA aneurysm (*N* = 3). All *in vivo* volunteers were recruited prospectively. The institutional review board approves all imaging studies, and written consent is obtained before scanning. All cardiovascular data are acquired using a 1.5 T MRI system (Siemens Avanto) with breathing navigator gating and prospective electrocardiogram triggering. Three ICA aneurysm data sets are acquired using a 3 T MRI system (Philips Achieva TX) with prospective cardiac triggering using a peripheral pulse unit. No contrast agent is used. Acquisition parameters for both types of data are listed in Table 1. For all datasets, Maxwell terms and gradient non-linearity are corrected during reconstruction. Eddy current phase offset is corrected offline.

**Table 1:** Acquisition parameters in our study for the synthetic and *in vivo* 4D-flow MRI data set.

	**Synthetic cerebrovascular**	***In vivo* Cerebrovascular**	***In vivo* Cardiovascular**
FOV [mm]	320–440 x 370–520 x 320–470	190 x 210 x 32	340–360 x 210–250 x 80–150
Acquisition matrix	-	128 x 128 x 32	160 x 100 x 32–64
Spatial res. [mm]	0.25 x 0.25 x 0.25	0.82 x 0.82 x 0.82	2.1–2.3 x 2.1–2.5 x 2.3–2.5
Temporal res. [ms]	36	55	40
Patient cohort	6	3	21
Scanner	-	3.0 T Philips Achieva TX	1.5 T Siemens avanto
TE/TR [ms]	-	2.9/4.6	2.54/5
*V*_*enc*_ [cm/s]	-	80	150
Parallel imaging	-	SENSE (*R* = 2) (Pruessmann et al., 1999)	PEAK-GRAPPA (*R* = 5) (Jung et al., 2008)
Cardiac gating	-	peripheral pulse unit	ECG

*FOV, field of view; TE, echo time; TR, repetition time; V_enc_, velocity encoding range; ECG, electrocardiogram*.

### 2.5. Data preparation

For synthetic and *in vivo* data sets, we rely on the following data preparation steps to create training data. We consider the acquired image volume as the high-resolution reference data.

1. We first convert the velocity data into phase using a *venc* = *v*_*max*_/π to avoid any phase warping. Next, we combine the magnitude with phase and transform the reference data into Fourier space. Note that we use synthetic data's segmentation mask as the dummy magnitude.
2. Then, we crop the low-frequency component from the k-space according to the downsampling factors.
3. We apply additive Gaussian noise with the k-space.
4. Finally, we apply Fourier inversion and multiply the phase with *venc* to obtain the low-resolution training data.

This process closely resembles the sub-sampling process in the MRI scanner (Gudbjartsson and Patz, 1995). For our training, we extract patches of sizes 24 × 24 × 12 × 3, 16 × 16 × 8 × 3, and 12 × 12 × 6 × 3 from the low-resolution volume for 2 ×, 3 ×, and 4 × super-resolution, respectively. The patches-size corresponding to the high-resolution volume is 48 × 48 × 24 × 3. The patches are selected on-the-fly during training from a random location of the training data. We normalize the input to [-1,1] by dividing the velocity with *v*_*max*_, the maximum velocity present.

### 2.6. Evaluation metrics

Peak velocity-to-noise ratio (PVNR) is commonly used to quantify the reconstruction quality of the estimated velocity field. We use PVNR as a primary metric to quantify the performance of our proposed super-resolution method. PVNR between reference (**u**) and the estimated velocity (**v**) is


(6) $$\begin{array}{rc} PVNR & =20\log_{10}\frac{1}{{\text{RMS}}_{vel}}\text{ dB} \end{array}$$

(7) $$\begin{array}{rc} {\text{RMS}}_{vel} & =\frac{1}{\underset{i}{\max}||{\text{u}}_{i}||}\sqrt{\frac{1}{N}\overset{N}{\underset{i=0}{\sum }}||{\text{u}}_{i}-{\text{v}}_{i}|{|}^{2}} \end{array}$$

where RMS_*vel*_ is the normalized-root-mean-squared-error of velocity. PVNR represents a combined error in the magnitude estimation and the phase estimation. Furthermore, we aim to deconstruct the source of error into its magnitude and phase component. As a measure of the error in magnitude estimation, we compare the normalized-root-mean-squared-error of speed (RMS_*speed*_) as described below


(8) $$\begin{array}{r} {\text{RMS}}_{speed}=\frac{1}{\underset{i}{\max}||{\text{u}}_{i}||}\sqrt{\frac{1}{N}\overset{N}{\underset{i=0}{\sum }}{\left(\left||{\text{u}}_{i}||-||{\text{v}}_{i}|\right|\right)}^{2}} \end{array}$$

We also compute Direction Error ($E_{dir}$) to measure the deviation of instantaneous velocity direction with respect to the reference velocity. The error in direction estimation is critical in some downstream tasks, such as streamline-tracing and path-line tracking, where the corresponding algorithm's accuracy depends on direction estimation accuracy.


(9) $$\begin{array}{r} E_{dir}=\frac{1}{N}\overset{N}{\underset{i=0}{\sum }}\left(1-\frac{\langle {\text{u}}_{i}\cdot {\text{v}}_{i}\rangle }{\left||{\text{u}}_{i}||||{\text{v}}_{i}|\right|}\right) \end{array}$$

Furthermore, we emphasize the flow consistency in terms of the flow divergence of the super-resolved flow field. We compute the root-mean-squared divergence in the region of interest and compare it against the high-resolution reference velocity.


(10) $$\begin{array}{r} {\text{RMS}}_{div}=\sqrt{\frac{1}{N}\overset{N}{\underset{i=0}{\sum }}|\nabla \cdot {\text{v}}_{i}{|}^{2}} \end{array}$$

## 3. Results

In this section, we describe our experiments and the main results. We refer to WDSR-3D as the 3D extension of WDSR by Yu et al. (2018). For the details of WDSR architecture, please refer to the original paper by Yu et al. (2018). This is one of the top-performing methods in the NTIRE super-resolution challenge. Hence, we select this as the baseline of our study and build our contribution upon it. We compare our work to the WDSR-3D for two main purposes. First, it is a strong baseline that is scalable to 3D. Second, its residual learning architecture is similar to the existing method (Ferdian et al., 2020) and provides a point of reference for comparison. The method by Ferdian et al. (2020) also requires magnitude images, which is not a good candidate for training with CFD simulated data. Since we are not using magnitude images in our training, we are unable to perform a direct comparison. Although it is not 100% identical, WDSR-3D is analogous to their method and can serve as a point of reference. In our experiment with WDSR-3D on synthetic data, we observe that ℓ_1_ loss offers on average 1dB PVNR improvement over ℓ_2_ loss for 2x super-resolution. From this observation, we chose WDSR-3D with ℓ_1_ loss as the baseline model for our experiments. We will denote SRflow (ℓ_1_), SRflow (mp-ℓ_1_), and SRflow (opt) trained with $J_{\ell_{1}}$, $J_{\text{mp-}\ell_{1}}$, and $J_{opt}$, respectively. The following two subsections (Experiment-1 & -2) are the descriptions of the experimental setup. For statistical significance analysis, we performed a Wilcoxon signed-rank test. For this, we collected predictions from all of the 3-fold validation. We declare statistical significance when the *p*-value is lower than 0.001. The analysis of their results is presented jointly in Section 4.

### 3.1. Experiment-1: Train on synthetic cerebrovascular data

First, we train and test our model on the synthetic cerebrovascular data. We perform three experiments with three different train-validation splits and report the combined results. This allows us to maximize training examples while performing required cross-validation. For each experiment, we have selected (120 samples) five subjects as training and the remaining one as the validation data (24 samples). The train-validation split was fixed across the models and loss functions for a fair comparison.

#### 3.1.1. Part A: Evaluation on synthetic cerebrovascular data

**Figure 5:**
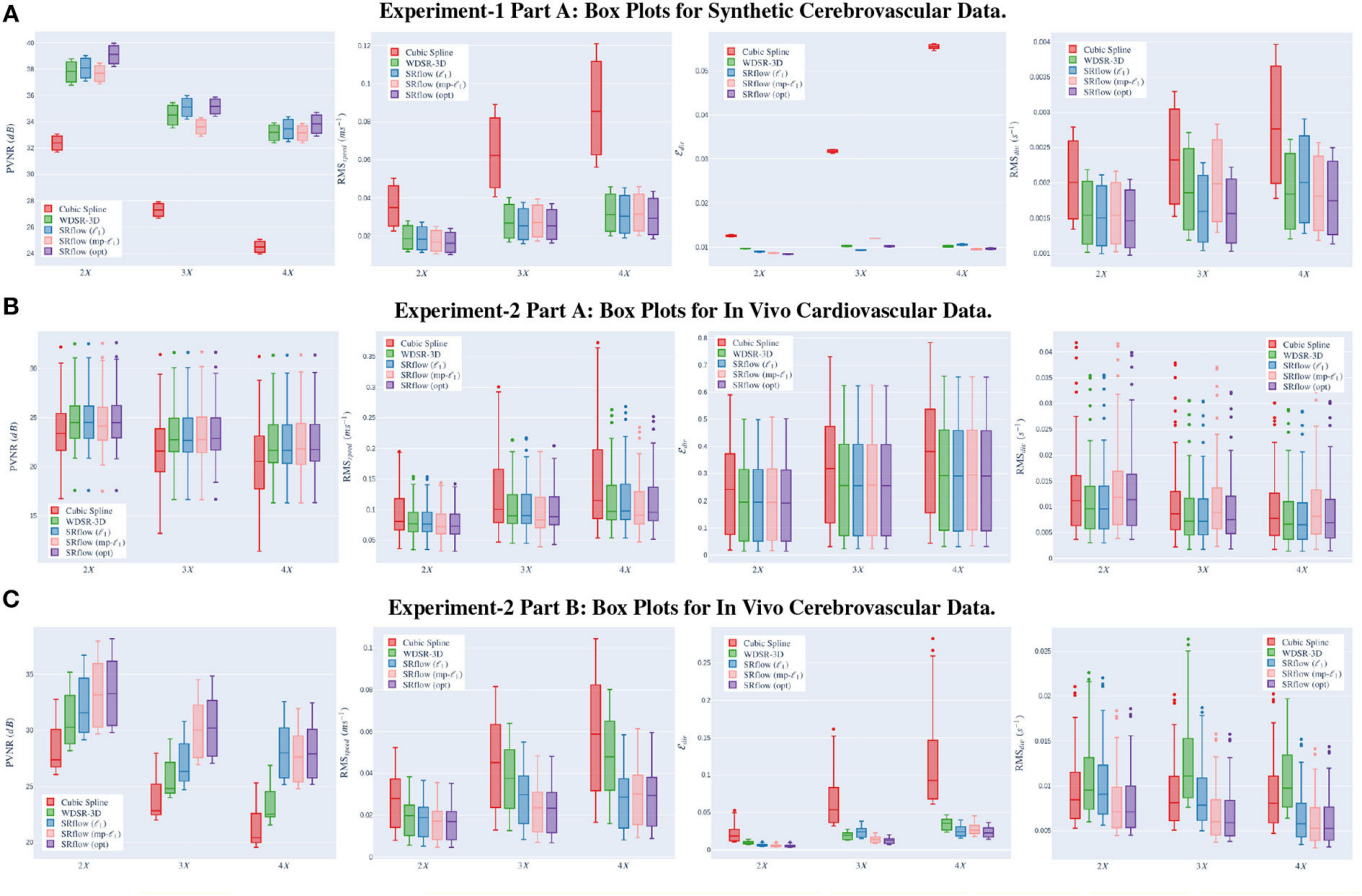
*Left*→*right* shows the box plots for peak velocity-to-noise ratio (PVNR), root-mean-squared-error (RMS)_*speed*_, $E_{dir}$, and RMS_*div*_, respectively. The x-axis shows different upscaling factors 2 ×, 3 ×, and 4 × respectively. Higher (↑) PVNR and lower (↓) RMS_*speed*_, $E_{dir}$ and RMS_*div*_ indicates better performance. Note that SRflow (opt) consistently outperforms the baseline models. **(A)** Experiment-1 Part A: box plots for synthetic cerebrovascular data. **(B)** Experiment-2 Part A: box plots for *in vivo* cardiovascular data. **(C)** Experiment-2 Part B: box plots for *in vivo* cerebrovascular data.

This experiment serves as the proof of concept for both our model and loss function. We compute each metric's mean and SD for the validation data over three independent trials. We train separate models for three different upscaling factors, such as 2 ×, 3 ×, and 4 × SR. Figure 5A shows the boxplot of four different metrics for the experiments on the synthetic data. We present the comparative result for different experiments set in Supplementary Table S1. Supplementary Figure S1 presents an exemplary temporal visualization of PVNR for a particular slice from the validation set. Supplementary Figure S2 presents the error profile of the velocity field for the corresponding slice location of the same example, which is done using Paraview.

#### 3.1.2. Part B: Evaluation on *in vivo* cerebrovascular data

We evaluate the model trained on synthetic cerebrovascular data on the 32 *in vivo* cerebrovascular samples from 2 subjects. Supplementary Table S2 shows the quantitative comparison of all the metrics for three different scaling factors. We observe that the improvement in metrics for scaling factors 2 × and 3 × is low compared to 4 ×. The improvement is also relatively lower than the improvement observed in Supplementary Table S1. Although SRflow (opt) consistently performs better than the cubic spline and baseline WDSR-3D, we investigate the inclusion of *in vivo* data during training in the following.

### 3.2. Experiment-2: Fine-tune on *in vivo* cardiovascular data

The previous experiment shows that the model trained on synthetic data does not offer the same degree of improvement over cubic-spline on *in vivo* cerebrovascular data for lower scaling factors. We attribute this to the fact that different noises and artifacts are present in the *in vivo* data (Johnson and Markl, 2010). We fine-tune the model using *in vivo* cardiovascular data to overcome this gap. We choose to fine-tune all our model Experiment-1 on *in vivo* cardiovascular data instead of *in vivo* cerebrovascular data because cardiovascular data have more samples than cerebrovascular data and consists of a significantly richer variation of noise and artifacts (Fathi et al., 2018). Similar to synthetic experiments, we perform three experiments with three different train-validation splits and report the combined results. For each experiment, we have selected 17 subjects as training and the remaining 4 as the validation set. Similar to before, the train-validation split was fixed across the models and loss functions for a fair comparison. Finally, we directly translate the trained model from cardiovascular data to *in vivo* aneurysm 4D-flow MRI data and evaluate the performance.

#### 3.2.1. Part A: Evaluation of *in vivo* cardiovascular data

**Figure 6:**
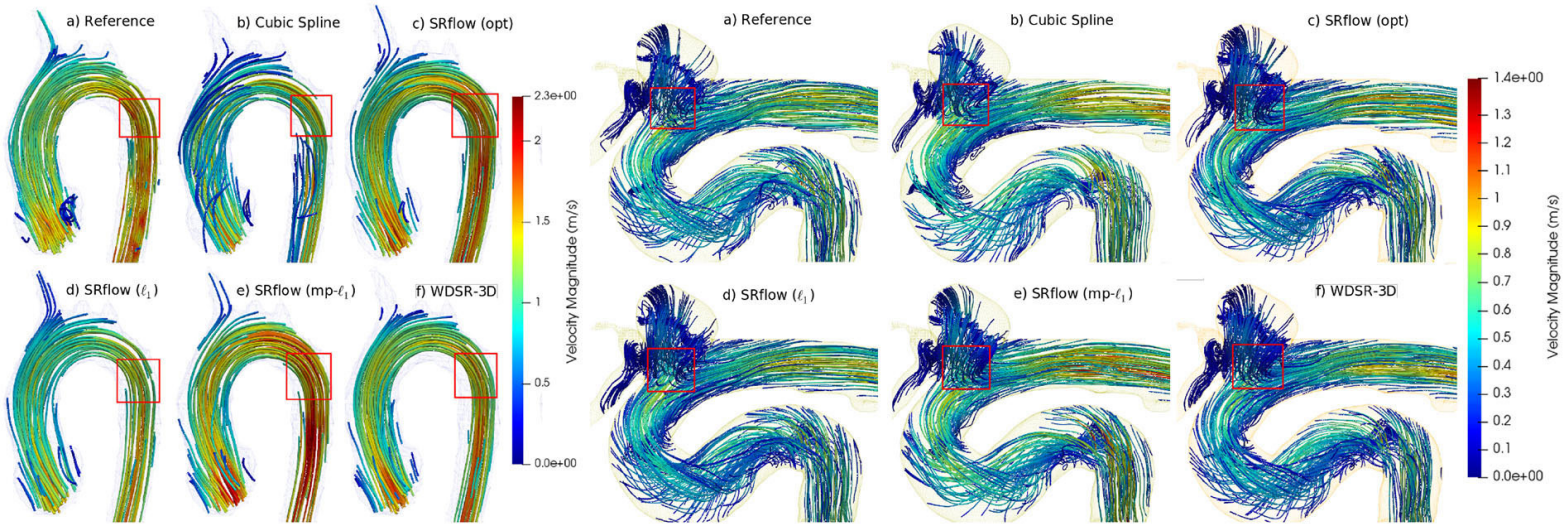
Qualitative comparison of streamlines obtained from different methods on two representative *in vivo* samples (left-cardiovascular and right-cerebrovascular). SRflow (opt) shows closer similarity to the reference streamlines for both the cases, while it reduces artifacts.

Similar to our synthetic data experiments, we perform the experiments for three different scenarios, such as 2 ×, 3 ×, and 4 × super-resolution. Figure 5B shows the boxplot of four different metrics for the validation data. The comparative result for this experiment is shown in Supplementary Table S3. Figure 6 shows a qualitative comparison of representative cardiac data from our experiments.

#### 3.2.2. Part B: Re-evaluation of *in vivo* cerebrovascular data

**Figure 7:**
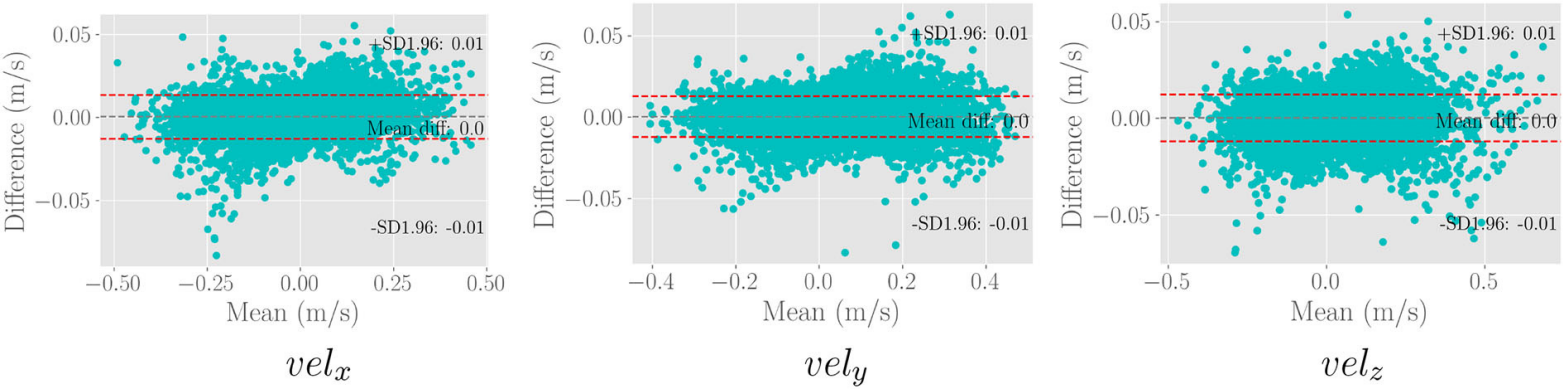
Bland-Altman plot for the three velocity components of 50,000 random samples from the *in vivo* cerebrovascular data for the SRflow(opt) model fine-tuned with cardiovascular data. We observe excellent agreement between the prediction and the reference.

**Figure 8:**
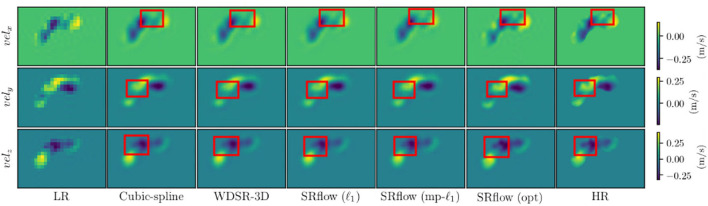
Qualitative images from different methods on a representative cerebrovascular sample. Note that cubic spline interpolation creates unnecessary amplification and attenuation of flow where SRflow (opt) preserves the flow intensity closest to the reference (HR).

We use the trained model from the cardiovascular experiments and evaluate the *in vivo* cerebrovascular data without further fine-tuning. Supplementary Table S4 shows the quantitative comparison of all the metrics for three different scaling factors. Figure 5C shows the boxplot of four different metrics for the *in vivo* cerebrovascular data. Figure 6 shows a qualitative comparison of representative aneurysm data from our experiments. Additionally, we have shown the Bland-Altman plot (Figure 7) for each velocity component, which shows good agreement with the reference, and the error is homogeneously scattered across the (mean ±1.96 × SD) range. Qualitative results from Figure 8 show that SRflow (opt) produces the closest prediction to the reference.

## 4. Discussion

### 4.1. Ablation study and transfer learning

#### 4.1.1. Effect of squeeze and excite block

From Figures 5A–C and Supplementary Tables S1–S4 in the Appendix, we observe that cubic-spline-based upsampling is consistently inferior compared to the learning-based solutions. The performance gain between these two increases with the upsampling factor for both the synthetic and *in vivo* data. However, the improvement for synthetic cerebrovascular data (Supplementary Table S1) is greater than the *in vivo* cardiovascular data (Supplementary Table S3). We attribute this to the ‘reference' 4D-flow MRI data being noisy, which may hamper the reconstruction quality measure. Furthermore, we notice that the SRflow (ℓ_1_) network achieves better PVNR, RMS_*speed*_, $E_{dir}$, and RMS_*div*_. than the WDSR-3D counterpart for all three cases of super resolutions 2 ×, 3 ×, and 4 ×. We find SRflow (ℓ_1_) results are statistically significant (*p* < 0.001) compared to WDSR-3D for all four metrics. We believe this is due to the fact that the diversity in feature space induced by the SE block better captures the inter-scale relationship of the velocity field.

#### 4.1.2. Effect of our proposed loss function (*mp-ℓ*_1_)

We investigate the effect of two different loss functions discussed in Section 2.2 on the SRflow architecture. For Experiment 1 (Supplementary Tables S1, S2), we see that SRflow (opt) performs consistently better than the other loss functions for all super-resolution factors concerning PVNR and RMS_*speed*_. For $E_{dir}$, SRflow (opt) produces the lowest error except 4 × factor in Supplementary Table S1. For RMS_*div*_, we observe that SRflow (ℓ_1_) produces the lowest error. We find the improvements from SRflow (opt) over baseline (WDSR-3D) and other SRflow variants to be statistically significant (*p* < 0.001) for PVNR, RMS_*speed*_, and $E_{dir}$. While we do not find any statistically significant (*p* > 0.001) difference between the SRflow (ℓ_1_) and SRflow (opt) for RMS_*div*_ for S1, the same is statistically significant (*p* < 0.001) for Supplementary Table S2.

For experiment 2 (Supplementary Tables S3, S4), SRflow (opt) improves PVNR for all scale factors compared to baseline (WDSR-3D) and other SRflow variants. For RMS_*speed*_, SRflow (opt) and SRflow (mp-ℓ_1_) result in the lowest error. For $E_{dir}$, SRflow (opt) produces the lowest error except for the 4 × factor in Supplementary Table S1. WDSR-3D and SRflow (ℓ_1_) produce the lowest RMS_*div*_. Although SRflow (opt) reduces $E_{dir}$ consistently, it produces slightly higher RMS_*speed*_ and RMS_*div*_ than SRflow (mp-ℓ_1_) and SRflow (ℓ_1_), respectively. We attribute this to the fact that mp-ℓ_1_ offers a trade-off between accurate magnitude and phase estimation of the velocity field during training on ‘noisy reference.' While higher PVNR ensures good signal quality for accurate quantitative analysis, lower $E_{dir}$ reduces error in the qualitative assessment, such as streamline tracing. We find the improvements from SRflow (opt) over baseline (WDSR-3D) and other SRflow variants to be statistically significant (*p* < 0.001) for PVNR, RMS_*speed*_, and $E_{dir}$. We find no statistically significant (*p* < 0.001) difference between the SRflow (ℓ_1_) and SRflow (opt) for RMS_*div*_ for Supplementary Tables S3, S4. In stark contrast with Supplementary Table S3, this shows the benefit of fine-tuning using *in vivo* data.

The mp-ℓ_1_ alone fails to improve the performance compared to the standard ℓ_1_, but it is evident from Supplementary Tables S1, S3 that when combined with the ℓ_1_ it outperforms both. We hypothesize that gradient from mp-ℓ_1_ loss is beneficial when the directional error is large because of the directional sensitivity, which is helpful for the ‘exploration' in the early stage of training. Additionally, since the loss curve of mp-ℓ_1_ is smoother compared to ℓ_1_ loss at lower error, ℓ_1_ provides a stronger gradient than mp-ℓ_1_, which is vital in the ‘exploitation' of the final stage of training. Hence, the performance improvement stands out when both loss functions are used simultaneously.

We experimented with divergence loss function, which can serve as a physics constrained regularizer. The divergence loss is defined below.


(11) $$\begin{array}{r} J_{\text{div}}=\frac{1}{n}\overset{n}{\underset{i=1}{\sum }}||\nabla \cdot {\text{v}}_{i}|{|}_{2} \end{array}$$

We have identical experiment settings for divergence loss as described before to have a fair comparison. We have observed from Table 2 that the inclusion of divergence loss improved the results in both cases. In particular, the divergence metric improved consistently across the different upsampling factors. We also observe that the gain is slightly higher in the case of SRflow (opt) than in standard ℓ_1_ loss, which reasserts the effectiveness of our proposed loss function. However, since divergence cannot be used as a standalone loss function, we conclude from these experiments that using it as a regularizer benefits the model.

**Table 2:** Effect of divergence loss and data augmentation strategy on synthetic cerebrovascular data.

**Methods**	***s***	**PVNR (dB) ↑**	**RMS_*speed*_ (*ms*^−1^) ↓**	**$E_{dir}$ ↓**	**RMS_*div*_ (*s*^−1^) ↓**
SRflow (opt)	×2	39.14 ± 0.629	0.0161 ± 0.00487	0.0084 ± 0.00008	0.0014 ± 0.00038
SRflow(ℓ_1_+*div*)	×2	38.89 ± 0.427	**0.0138 ± 0.00346**	**0.0081 ± 0.00005**	**0.0012 ± 0.00026**
SRflow (opt+mixed data)	×2	39.21 ± 0.301	**0.0138 ± 0.00249**	0.0084 ± 0.00008	0.0013 ± 0.00028
SRflow (opt+*div*)	×2	**38.93 ± 0.426**	**0.0138 ± 0.00342**	0.0084 ± 0.00007	**0.0012 ± 0.00027**
SRflow (opt)	×3	35.20 ± 0.520	0.0253 ± 0.00732	0.0102 ± 0.00008	0.0015 ± 0.00042
SRflow(ℓ_1_+*div*)	×3	35.45 ± 0.376	0.0205 ± 0.00494	**0.0088 ± 0.00006**	0.0013 ± 0.00029
SRflow (opt+mixed data)	×3	35.19 ± 0.260	0.0241 ± 0.00405	0.0102 ± 0.00005	0.0015 ± 0.00026
SRflow (opt+*div*)	×3	**35.94 ± 0.389**	**0.0196 ± 0.00478**	**0.0088 ± 0.00006**	**0.0012 ± 0.00028**
SRflow (opt)	×4	33.87 ± 0.642	0.0293 ± 0.00888	0.0097 ± 0.00015	0.0017 ± 0.00048
SRflow(ℓ_1_+*div*)	×4	34.44 ± 0.399	**0.0226 ± 0.00560**	**0.0096 ± 0.00006**	0.0014 ± 0.00032
SRflow (opt+mixed data)	×4	33.24 ± 0.256	0.0269 ± 0.00603	**0.0096 ± 0.00002**	**0.0013 ± 0.00030**
SRflow (opt+*div*)	×4	**34.51 ± 0.428**	0.0229 ± 0.00573	0.0098 ± 0.00003	**0.0013 ± 0.00031**

*The bold values mean the highest score of the corresponding scale factor ×2, ×3, and ×4*.

#### 4.1.3. Transfer learning

We fine-tune our model on the *in vivo* cardiovascular data and validate it on the *in vivo* cerebrovascular data, scanned in an entirely different scanner and velocity encoding value. Despite the difference in the anatomical region, we observe that our proposed SRflow (opt) improves all metrics significantly (c.f. Supplementary Table S4). Especially we observe that it produces the lowest RMS_*div*_ compared to other methods, which is also statistically significant. This observation confirms that our model can be seamlessly transferred to other existing MRI acquisition configurations without the need for any further local fine-tuning.

We have performed additional experiments comprising joint CFD and *in vivo* datasets and reported Tables 2, 3. We compare the result from joint training and training sequentially in synthetic and *in vivo* data on both synthetic and *in vivo* data. We observe a marginal improvement on the synthetic test set. However, the results deteriorate slightly on the *in vivo* test set. We attribute this to the fact that the MRI artifacts and noise are difficult to model in the CFD data.

**Table 3:** Comparison on data augmentation strategy on *in vivo* cerebrovascular data.

**Methods**	***s***	**PVNR (dB) ↑**	**RMS_*speed*_ (*ms*^−1^) ↓**	**$E_{dir}$ ↓**	**RMS_*div*_ (*s*^−1^) ↓**
SRflow (opt)	×2	**33.52 ± 2.703**	**0.0164 ± 0.00878**	0.0053 ± 0.00160	**0.0083 ± 0.00394**
SRflow (opt+mixed data)	×2	33.32 ± 2.588	0.0167 ± 0.00878	**0.0051 ± 0.00131**	0.0083 ± 0.00387
SRflow (opt)	×3	**30.46 ± 2.473**	**0.0228 ± 0.01188**	**0.0120 ± 0.00345**	**0.0070 ± 0.00333**
SRflow (opt+mixed data)	×3	30.39 ± 2.390	0.0229 ± 0.01177	0.0122 ± 0.00347	0.0070 ± 0.00336
SRflow (opt)	×4	**28.30 ± 2.321**	**0.0279 ± 0.01456**	**0.0242 ± 0.00723**	0.0067 ± 0.00325
SRflow (opt+mixed data)	×4	28.03 ± 2.258	0.0294 ± 0.01491	0.0245 ± 0.00694	**0.0062 ± 0.00311**

*The bold values mean the highest score of the corresponding scale factor ×2, ×3, and ×4*.

### 4.2. Global quality evaluation of reconstructed velocity

Besides the voxel-wise reconstruction metrics, analyzing the effect of the super-resolved velocity field on a global level, such as path integration along the flow field, are also important. We compare the computed streamlines for *in vivo* 4D-flow MRI data to assess the global reconstruction quality.

**Figure 9:**
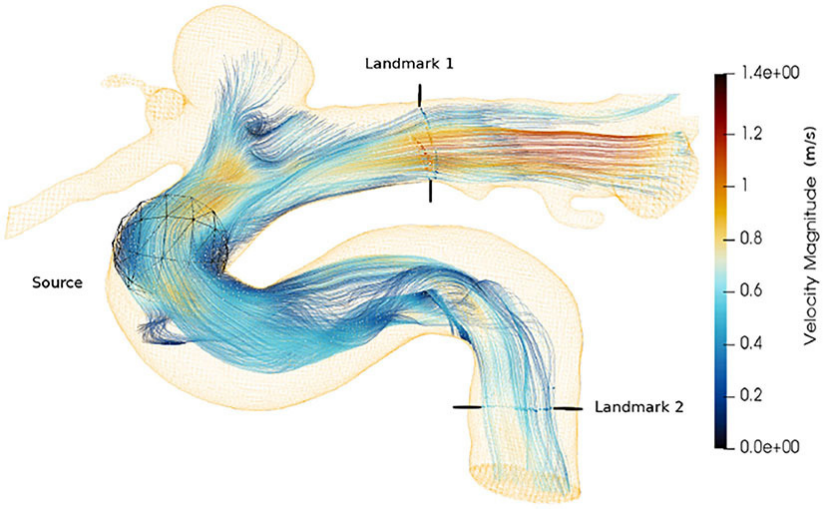
Velocity streamlines from an *in vivo* cerebrovascular 4D-flow MRI. The seed points are placed using a spherical source. The landmarks are used in Table 4.

Streamlines (Cebral et al., 2011) is an important visualization technique, often used as the primary mode for clinicians' interpretable representation of 4D-flow MRI. The continuity of streamlines can be used as an alternative way to measure the quality of the super-resolved vector fields. We start the streamline tracing near the aneurysm with 2000 seed points and extend toward both the landmarks as shown in Figure 9. We compute the relative error between the number of streamlines produced by each super-resolver volume and the reference streamline. Table 4 shows the mean of the relative error over one cardiac cycle at two landmarks for the 4 × super-resolution task. We find that the cubic spline always underestimates the number of streamlines, and SRflow (opt) produces the lowest relative errors.

**Table 4:** The mean of relative error in the number of streamlines with respect to the reference *in vivo* data over one cardiac cycle, which is computed at two landmarks as shown in Figure 9 for different super-resolution methods.

**Method**	**Landmark 1**	**Landmark 2**
Cubic-spline	0.66	0.76
WDSR-3D	0.50	0.16
SRflow(ℓ_1_)	0.37	0.16
SRflow (mp-ℓ_1_)	0.36	0.21
SRflow (opt)	0.31	0.11

### 4.3. Runtime comparison

We compare the runtime for a 96 × 132 × 48 3D volume between the cubic spline and the neural network-based model for three different resolutions. We compare the runtime in a workstation equipped with Intel(R) Xeon(R) W-2123 and 64 GB DDR4 RAM. The comparison is shown in Table 5. SRflow offers significant computational speedup, which is favorable for clinical application.

**Table 5:** Runtime comparison between SRflow and cubic spline, where we see that SRflow is much faster.

**Method**	**2x**	**3x**	**4x**
Cubic spline	76.42 s	76.19 s	73.09 s
SRflow (opt)	8.17 s	3.80 s	2.86 s

### 4.4. Limitation and outlook

While the improved resolution will be beneficial in increased stability for numerical gradient computation, its accuracy is still limited to finite difference schemes and the maximum super-resolution factor learned during training for optimal performance. Furthermore, the current study is limited in exploring different spatial super-resolution factors, and temporal super-resolution is of future research interest. Additionally, including other realistic perturbations, such as scan-rescan variability, phase aliasing, and eddy current effect, would be of interest to include in the model. Future research will include increasing the number of samples of the *in vivo* cohort. Future work will also focus on further quantitative assessment of advanced parameters, such as WSS and KE.

## 5. Conclusion

This paper investigates the effectiveness of deep learning in super-resolving 4D-flow MRI data up to 4x resolution. We have started with a strong baseline model and gradually improved it by incorporating expressive squeeze and excite block. Furthermore, we introduce a novel robust loss function with directional sensitivity suitable for velocity data. With extensive validation, we demonstrated the effectiveness of the introduced component. Next, we show that the model learned from synthetic CFD data still requires finetuning on *in vivo* data for improved performance. Importantly, this finetuning is not dataset-dependant and can be applied seamlessly to other *in vivo* datasets without further finetuning. Naturally, we have improved runtime compared to the classical interpolation method, which could enable future 4D-flow MRI acquisitions at lower resolution—and thus with decreased scan time—without compromising the accuracy of quantitative flow analysis.

## Data availability statement

The datasets presented in this article are not readily available because restrictions apply to the sharing of patient data that supports the findings of this study. Requests to access the datasets should be directed to SS, suprosanna.shit@tum.de.

## Ethics statement

The studies involving human participants were reviewed and approved by Institutional Ethics Committee, TUM. The patients/participants provided their written informed consent to participate in this study.

## Author contributions

SS and JZ conceived and designed the study and performed the experiments. JZ and AS acquired the datasets. AS simulated the synthetic blood flow. SS, JZ, and AS preprocessed the dataset. SS, JZ, IE, JP, and CP analyzed the results. SS, JZ, CP, and BM prepared the manuscript. BM supervised the study. All authors contributed to the article and approved the submitted version.

## Funding

SS and IE are supported by the Translational Brain Imaging Training Network under the EU Marie Sklodowska-Curie programme (Grant ID: 765148).

## Conflict of interest

The authors declare that the research was conducted in the absence of any commercial or financial relationships that could be construed as a potential conflict of interest.

## Publisher's note

All claims expressed in this article are solely those of the authors and do not necessarily represent those of their affiliated organizations, or those of the publisher, the editors and the reviewers. Any product that may be evaluated in this article, or claim that may be made by its manufacturer, is not guaranteed or endorsed by the publisher.
