# Supplementary material for: SRflow: Deep learning based super-resolution of 4D-flow MRI data
Source: Front Artif Intell. 2022 Aug 12;5:928181. doi: 10.3389/frai.2022.928181 (PMC9411720; doi:10.3389/frai.2022.928181)
Supplement: Supplementary file 1 [file Data_Sheet_1.pdf]

Supplementary Material: Deep Learning Based Super Resolution of 4D-flow MRI Data

1 QUANTITATIVE RESULT

Methods	s	PVNR (dB) ↑	RMS$_{speed}$ (ms^{-1}) ↓	$\mathcal{E}_{dir}$ ↓	RMS$_{div}$ (s^{-1}) ↓
Cubic Spline	×2	32.42 ± 0.491	0.0348 ± 0.00987	0.0126 ± 0.00017	0.0019 ± 0.00051
WDSR-3D	×2	37.84 ± 0.705	0.0187 ± 0.00578	0.0097 ± 0.00005	0.0015 ± 0.00041
SRflow (ℓ_1)	×2	38.12 ± 0.696	0.0182 ± 0.00563	0.0090 ± 0.00009	**0.0014 ± 0.00039**
SRflow (mp-ℓ_1)	×2	37.71 ± 0.553	0.0168 ± 0.00508	0.0086 ± 0.00008	0.0015 ± 0.00040
SRflow (opt)	×2	**39.14 ± 0.629**	**0.0161 ± 0.00487**	**0.0084 ± 0.00008**	**0.0014 ± 0.00038**
Cubic Spline	×3	27.33 ± 0.444	0.0621 ± 0.01720	0.0317 ± 0.00030	0.0023 ± 0.00063
WDSR-3D	×3	34.55 ± 0.682	0.0269 ± 0.00826	**0.0102 ± 0.00008**	0.0018 ± 0.00054
SRflow (ℓ_1)	×3	**35.15 ± 0.634**	**0.0254 ± 0.00767**	0.0093 ± 0.00008	**0.0015 ± 0.00044**
SRflow (mp-ℓ_1)	×3	34.64 ± 0.495	0.0271 ± 0.00786	0.0119 ± 0.00002	0.0019 ± 0.00054
SRflow (opt)	×3	**35.20 ± 0.520**	**0.0253 ± 0.00732**	**0.0102 ± 0.00008**	**0.0015 ± 0.00042**
Cubic Spline	×4	24.53 ± 0.394	0.0851 ± 0.02302	0.0554 ± 0.00049	0.0027 ± 0.00077
WDSR-3D	×4	33.22 ± 0.540	0.0313 ± 0.00914	0.0102 ± 0.00012	0.0018 ± 0.00050
SRflow (ℓ_1)	×4	33.50 ± 0.676	0.0304 ± 0.00930	0.0105 ± 0.00014	0.0020 ± 0.00057
SRflow (mp-ℓ_1)	×4	33.18 ± 0.520	0.0315 ± 0.00910	**0.0095 ± 0.00012**	0.0018 ± 0.00049
SRflow (opt)	×4	**33.87 ± 0.642**	**0.0293 ± 0.00888**	**0.0097 ± 0.00015**	**0.0017 ± 0.00048**

Table S1: Experiment-1 Part A: Synthetic Cerebrovascular Results

Methods	s	PVNR (dB) ↑	RMS$_{speed}$ (ms^{-1}) ↓	$\mathcal{E}_{dir}$ ↓	RMS$_{div}$ (s^{-1}) ↓
Cubic Spline	×2	28.37 ± 2.046	0.0274 ± 0.01348	0.0228 ± 0.01275	0.0096 ± 0.00439
WDSR-3D	×2	29.33 ± 2.227	0.0248 ± 0.01260	0.0220 ± 0.00579	**0.0071 ± 0.00347**
SRflow (ℓ_1)	×2	29.45 ± 2.202	0.0245 ± 0.01246	0.0209 ± 0.00625	**0.0068 ± 0.00329**
SRflow (mp-ℓ_1)	×2	30.01 ± 2.215	0.0226 ± 0.01148	0.0182 ± 0.00490	0.0072 ± 0.00341
SRflow (opt)	×2	**30.56 ± 2.393**	**0.0220 ± 0.01149**	**0.0146 ± 0.00403**	0.0072 ± 0.00346
Cubic Spline	×3	23.81 ± 1.831	0.0447 ± 0.02161	0.0684 ± 0.03950	0.0092 ± 0.00422
WDSR-3D	×3	26.21 ± 1.809	0.0334 ± 0.01628	0.0525 ± 0.02257	0.0071 ± 0.00353
SRflow (ℓ_1)	×3	26.99 ± 1.923	0.0312 ± 0.01544	0.0425 ± 0.01636	**0.0064 ± 0.00330**
SRflow (mp-ℓ_1)	×3	26.80 ± 2.020	0.0314 ± 0.01555	0.0425 ± 0.01318	0.0070 ± 0.00352
SRflow (opt)	×3	**27.36 ± 2.014**	**0.0300 ± 0.01489**	**0.0367 ± 0.01250**	0.0067 ± 0.00350
Cubic Spline	×4	21.31 ± 1.738	0.0583 ± 0.02795	0.1214 ± 0.06776	0.0091 ± 0.00437
WDSR-3D	×4	25.15 ± 1.637	0.0368 ± 0.01760	0.0738 ± 0.03595	0.0068 ± 0.00341
SRflow (ℓ_1)	×4	**25.55 ± 1.736**	0.0359 ± 0.01733	**0.0616 ± 0.02935**	**0.0063 ± 0.00331**
SRflow (mp-ℓ_1)	×4	25.08 ± 1.835	0.0370 ± 0.01802	0.0677 ± 0.02744	0.0068 ± 0.00352
SRflow (opt)	×4	**25.61 ± 1.848**	**0.0354 ± 0.01740**	**0.0611 ± 0.02672**	0.0066 ± 0.00352

Table S2: Experiment-1 Part B: In Vivo Cerebrovascular 4D-flow MRI Results

Performance comparison of our proposed method with the baseline model and cubic-spline-based interpolation. We compare three different loss functions in our study for the proposed network to investigate contributions each of its contributions to the vector-field super-resolution. Higher (↑) PVNR and lower (↓) RMS$_{speed}$, $\mathcal{E}_{dir}$ and RMS$_{div}$ indicates better performance. We pairwise report Wilcoxon signed rank

between the best performing methods (shown in bold) and the other methods for all the metrics. Methods that do not differ significantly from the best performing one (p-value> 0.001), are also reported in bold.

**Methods**	s	**PVNR (dB)** $\uparrow$	**RMS**$_{speed}$ $(ms^{-1}) \downarrow$	$\mathcal{E}_{dir} \downarrow$	**RMS**$_{div}$ $(s^{-1}) \downarrow$
Cubic Spline	$\times 2$	23.53 ± 3.009	0.0936 ± 0.03924	0.2316 ± 0.15496	0.0131 ± 0.00872
WDSR-3D	$\times 2$	24.80 ± 2.477	0.0805 ± 0.02708	$\mathbf{0.1902 \pm 0.13379}$	$\mathbf{0.0113 \pm 0.00741}$
SRflow (ℓ_1)	$\times 2$	$\mathbf{24.82 \pm 2.481}$	0.0805 ± 0.02696	$\mathbf{0.1898 \pm 0.13372}$	$\mathbf{0.0113 \pm 0.00745}$
SRflow (mp-ℓ_1)	$\times 2$	24.81 ± 2.666	$\mathbf{0.0762 \pm 0.02561}$	0.1929 ± 0.13352	0.0136 ± 0.00872
SRflow (opt)	$\times 2$	$\mathbf{24.86 \pm 2.532}$	$\mathbf{0.0760 \pm 0.02542}$	$\mathbf{0.1892 \pm 0.13317}$	0.0130 ± 0.00835
Cubic Spline	$\times 3$	21.60 ± 3.642	0.1252 ± 0.06540	0.3096 ± 0.18966	0.0108 ± 0.00825
WDSR-3D	$\times 3$	23.17 ± 2.774	0.1016 ± 0.03806	0.2495 ± 0.16765	$\mathbf{0.0090 \pm 0.00663}$
SRflow (ℓ_1)	$\times 3$	23.16 ± 2.784	0.1021 ± 0.03857	0.2485 ± 0.16736	$\mathbf{0.0090 \pm 0.00659}$
SRflow (mp-ℓ_1)	$\times 3$	23.15 ± 2.865	$\mathbf{0.0949 \pm 0.03669}$	0.2499 ± 0.16787	0.0109 ± 0.00788
SRflow (opt)	$\times 3$	$\mathbf{23.26 \pm 2.735}$	$\mathbf{0.0983 \pm 0.03621}$	$\mathbf{0.2482 \pm 0.16734}$	$\mathbf{0.0094 \pm 0.00694}$
Cubic Spline	$\times 4$	20.55 ± 4.061	0.1476 ± 0.08548	0.3609 ± 0.20501	0.0100 ± 0.00822
WDSR-3D	$\times 4$	22.27 ± 3.031	0.1156 ± 0.04693	0.2865 ± 0.18165	$\mathbf{0.0082 \pm 0.00633}$
SRflow (ℓ_1)	$\times 4$	22.25 ± 3.065	0.1168 ± 0.04817	$\mathbf{0.2845 \pm 0.18102}$	$\mathbf{0.0082 \pm 0.00626}$
SRflow (mp-ℓ_1)	$\times 4$	$\mathbf{22.29 \pm 3.076}$	$\mathbf{0.1061 \pm 0.04399}$	0.2869 ± 0.18090	0.0102 ± 0.00782
SRflow (opt)	$\times 4$	$\mathbf{22.38 \pm 2.963}$	0.1115 ± 0.04442	$\mathbf{0.2843 \pm 0.18081}$	$\mathbf{0.0086 \pm 0.00663}$

Table S3: Experiment-2 Part A: In Vivo Cardiovascular 4D-flow MRI Results

**Methods**	s	**PVNR (dB)** $\uparrow$	**RMS**$_{speed}$ $(ms^{-1}) \downarrow$	$\mathcal{E}_{dir} \downarrow$	**RMS**$_{div}$ $(s^{-1}) \downarrow$
Cubic Spline	$\times 2$	28.37 ± 2.046	0.0274 ± 0.01348	0.0228 ± 0.01275	0.0096 ± 0.00439
WDSR-3D	$\times 2$	30.93 ± 2.155	0.0191 ± 0.00948	0.0096 ± 0.00184	0.0107 ± 0.00460
SRflow (ℓ_1)	$\times 2$	32.20 ± 2.373	0.0182 ± 0.00912	0.0062 ± 0.00147	0.0102 ± 0.00456
SRflow (mp-ℓ_1)	$\times 2$	33.38 ± 2.678	0.0166 ± 0.00885	0.0057 ± 0.00149	$\mathbf{0.0083 \pm 0.00388}$
SRflow (opt)	$\times 2$	$\mathbf{33.52 \pm 2.703}$	$\mathbf{0.0164 \pm 0.00878}$	$\mathbf{0.0053 \pm 0.00160}$	$\mathbf{0.0083 \pm 0.00394}$
Cubic Spline	$\times 3$	23.81 ± 1.831	0.0447 ± 0.02161	0.0684 ± 0.03950	0.0092 ± 0.00422
WDSR-3D	$\times 3$	25.59 ± 1.628	0.0373 ± 0.01592	0.0190 ± 0.00450	0.0127 ± 0.00520
SRflow (ℓ_1)	$\times 3$	27.03 ± 1.855	0.0289 ± 0.01374	0.0244 ± 0.00684	0.0089 ± 0.00378
SRflow (mp-ℓ_1)	$\times 3$	30.23 ± 2.373	0.0231 ± 0.01187	0.0139 ± 0.00393	$\mathbf{0.0070 \pm 0.00336}$
SRflow (opt)	$\times 3$	$\mathbf{30.46 \pm 2.473}$	$\mathbf{0.0228 \pm 0.01188}$	$\mathbf{0.0120 \pm 0.00345}$	$\mathbf{0.0070 \pm 0.00333}$
Cubic Spline	$\times 4$	21.31 ± 1.738	0.0583 ± 0.02795	0.1214 ± 0.06776	0.0091 ± 0.00437
WDSR-3D	$\times 4$	27.82 ± 2.192	0.0296 ± 0.01497	0.0281 ± 0.00772	$\mathbf{0.0062 \pm 0.00306}$
SRflow (ℓ_1)	$\times 4$	28.22 ± 2.271	0.0288 ± 0.01463	$\mathbf{0.0236 \pm 0.00669}$	$\mathbf{0.0063 \pm 0.00310}$
SRflow (mp-ℓ_1)	$\times 4$	27.31 ± 1.501	$\mathbf{0.0277 \pm 0.01978}$	0.0346 ± 0.00749	0.0111 ± 0.00468
SRflow (opt)	$\times 4$	$\mathbf{28.30 \pm 2.321}$	$\mathbf{0.0279 \pm 0.01456}$	$\mathbf{0.0242 \pm 0.00723}$	$\mathbf{0.0067 \pm 0.00325}$

Table S4: Experiment-2 Part B: In Vivo Cerebrovascular 4D-flow MRI Results

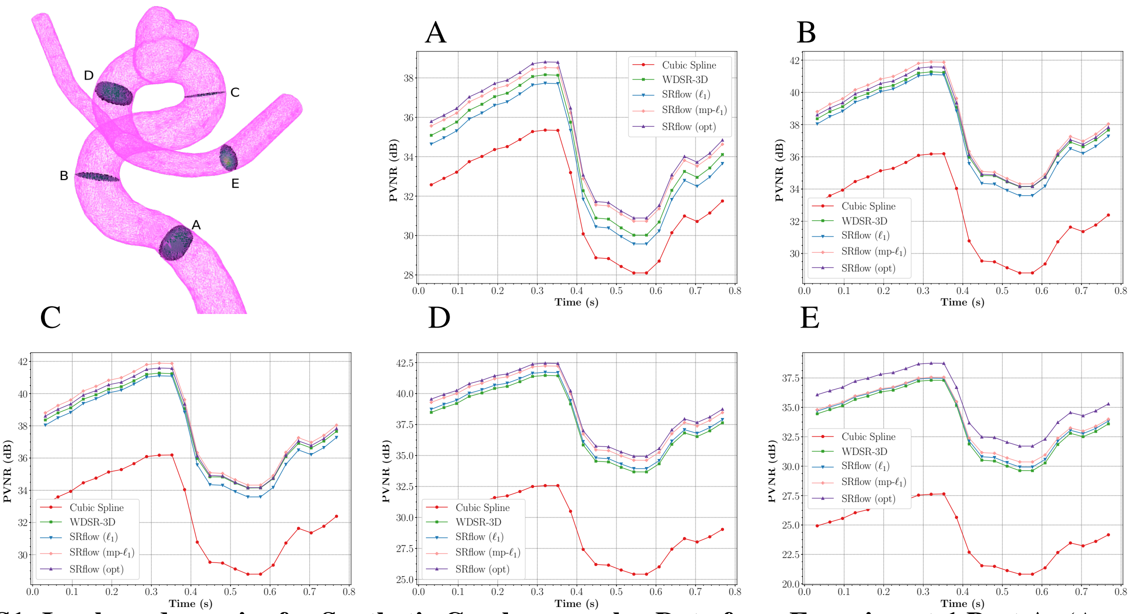

Figure S1: In-plane dynamics for Synthetic Cerebrovascular Data from Experiment-1 Part A: (A $\rightarrow$ E) shows the one cardiac cycle dynamics for PVNR for corresponding slices (A $\rightarrow$ E) of the aneurysm geometry, respectively, for the upscaling factor of $2\times$. All learning-based solutions outperform cubic-spline based super-resolution. SRflow (opt) and SRflow ($mp - \ell_1$) produces the best score in all 5 cases.

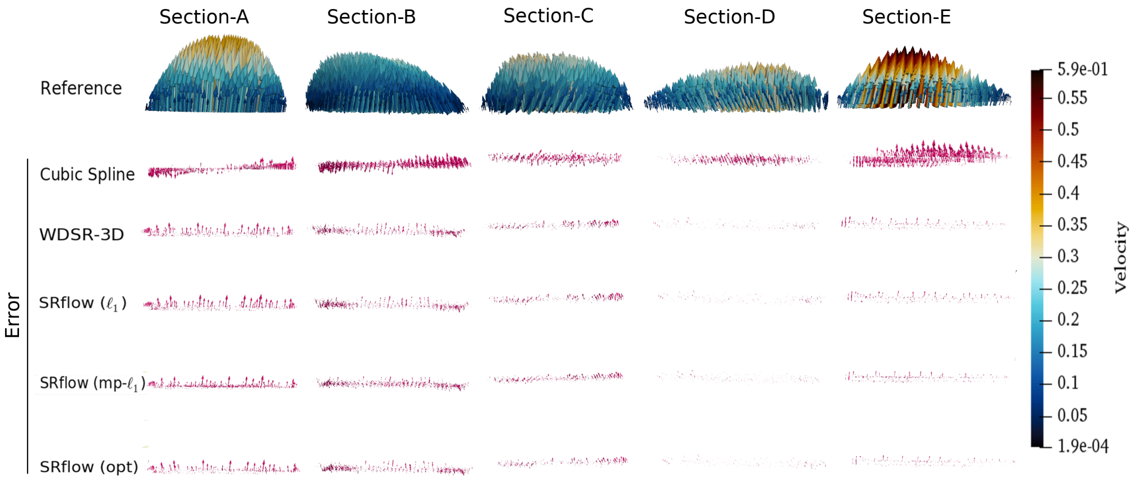

Figure S2: Flow Profile for Synthetic Cerebrovascular Data from Experiment-1 Part A: The first row shows the velocity profile of the reference data at the peak systolic time for five different cross-sections, as shown in Fig S1. The subsequent rows show the error in the velocity profile for different predictions. We observe that the cubic spline has a significant amount of error, and SRflow (opt) creates the least amount of error for all five cross-sections.
